# A visualized left‐sided accessory pathway away from the mitral annulus using open window mapping with the early meets late algorithm

**DOI:** 10.1002/joa3.12910

**Published:** 2023-09-04

**Authors:** Yuka Taguchi, Junya Hosoda, Shuichi Miyagawa, Akira Horigome, Toshiyuki Ishikawa

**Affiliations:** ^1^ Department of Cardiology Yokohama City University Graduate School of Medicine Yokohama city Kanagawa Japan

**Keywords:** atrioventricular reentry tachycardia, orthodromic reciprocating tachycardia, supraventricular tachycardia, total activation mapping, Wolff Parkinson white syndrome

A 70‐year‐old woman who presented with intermittent palpitation symptoms for the past few years. There was no pre‐excitation during sinus rhythm on 12‐lead surface ECG and a Holter ECG showed supraventricular tachycardia (SVT) (Figure 1A). After informed consent was obtained, catheter ablation was performed.

**FIGURE 1:**
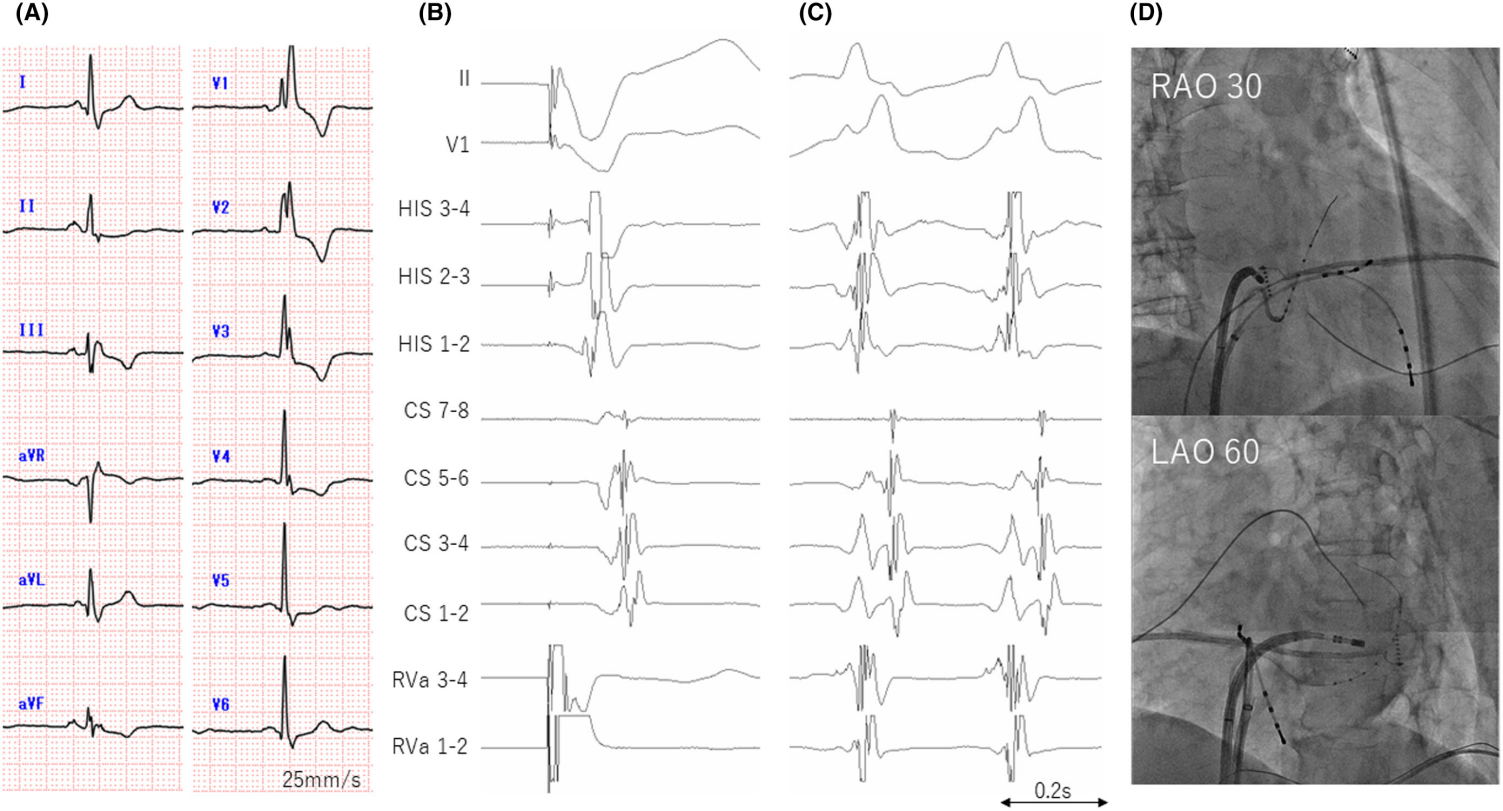
A: 12‐lead ECG before ablation showed sinus rhythm and right bundle branch block without antegrade pre‐excitation. Intracardiac electrograms during RVa pacing in B, and SVT in C. Early atrial excitation during RVa pacing was recorded in CS5‐6, identical to that during SVT. D: Catheter position at the successful ablation site in RAO and LAO views. CS, coronary sinus; HIS, His bundle; LAO, left anterior oblique; RAO, right anterior oblique; RVa, right ventricular apex; SVT, supraventricular tachycardia.

Early atrial excitation during right ventricular (RV) apex pacing was recorded in the left posterolateral wall (Figure 1B), showing an intermittent block and no decremental property. SVT was induced by an atrial double‐coupled extra stimulus with a cycle length of 270 ms, and early atrial excitation was identical to that during RV pacing (Figure 1B,C). Because of non‐sustained status, electrophysiologic studies during tachycardia were not available, but based on other findings, SVT was diagnosed as orthodromic reciprocating tachycardia (ORT) via a concealed left posterolateral AP.

To identify the location of the intermittent retrograde AP conduction, the open window mapping (OWM) during RV apex pacing was performed via a trans‐septal approach by using a multipolar catheter and three‐dimensional mapping system (Pentaray, CARTO3; Biosense Webster, Inc, Irvine, CA). The reference was set to the pacing spike, and the window of interest was set from +20 ms to +200 ms to include ventricular to atrial potentials. Local ventricular and atrial potentials around the posterolateral wall of the mitral annulus (MA) were acquired using Pentaray. These potentials are annotated on the wavefront where the distal electrode's steepest unipolar negative dV/dt coincides with the bipolar downslope. Early Meets Late (EML) displays a white line when there is a time‐phase difference between any two adjacent points showing the activation delay between the ventricle and the atrium, which could be regarded as an atrioventricular annulus. The lower threshold was adjusted to 30% to match the propagation map.

A typical AP location is shown as a white line gap where the ventricular and atrial potentials are continuous. However, there was no white line defect, and the earliest atrial activation site (EAAS) was located 2 cm above the MA (Figure 2A). The propagation map showed that after the ventricular excitation by RV pacing conducted to the MA, the excitation temporarily disappeared, and propagated as if it was springing from the left atrial posterolateral wall (Figure 3, Supplemental Movie S1). Atrial excitation on the MA was apparently delayed and no AP potential was observed. Radiofrequency applications to the EAAS area were performed. The power output was 30 W and the average contact force was 11–15 g. The effective sites where intermittent AP conduction blockade was achieved, and the successful site required over 5–10 s of radiofrequency application. Each application continued for 30–50 s. The EAAS subtly sifted to the adjacent region after effective applications and then multiple times ablation to the wider area was required to eliminate the AP on the epicardial side (Figure 2B). SVT has not recurred for more than 1 year.

**FIGURE 2:**
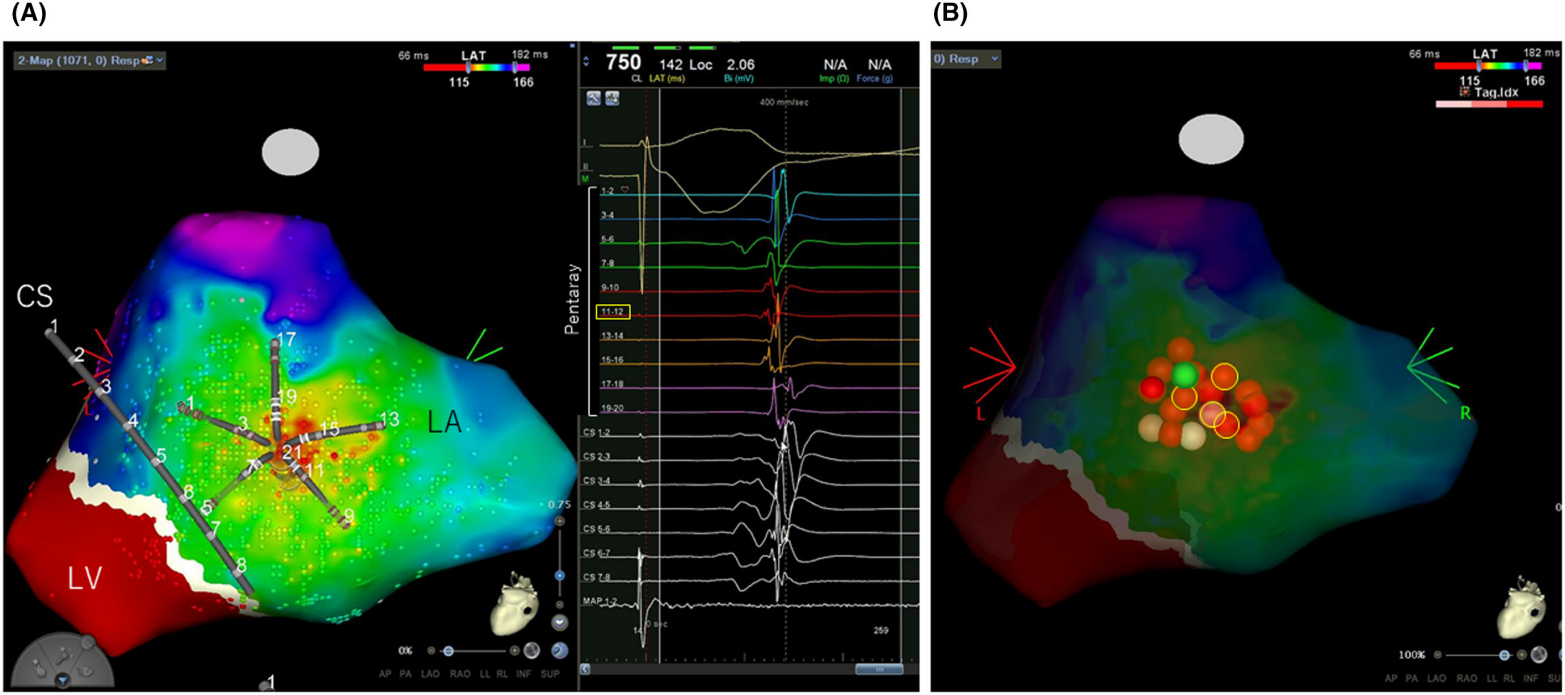
A: Open window mapping with Early Meets Late (EML) during RVa pacing from the left posterolateral view. Local ventricular and atrial potentials around the posterolateral wall of the mitral annulus (MA) were acquired using Pentaray via a transseptal approach. The potentials are annotated on the wavefront where the steepest unipolar ‐dV/dt of the distal electrode coincides with the bipolar downslope. EML was set by 30% of the lower threshold and 90% of the upper threshold to match the propagation map, showing a white line that indicates the MA. Notably, there was no white line gap, and the activation map showed that the earliest atrial activation site (EAAS) was located 2 cm above the white line. The intracardiac electrogram showed that atrial excitation in CS5‐6 was apparently delayed from the EAAS corresponding to 11–12 poles of Pentaray (yellow square) at which no ventricular potential was observed. B: Ablation sites to the EAAS area. Tags in a yellow frame showed effective sites leading to an intermittent retrograde accessory pathway (AP) conduction block during RVa pacing. A green tag showed the successful site requiring a radiofrequency application for more than 10 s to eliminate atrial attachment of the AP on the epicardial side. Others are unsuccessful or additional ablation sites. White tags indicate ablation index values lower than 400, light red tags indicate above 400, and dark red tags above 450. The area of effective and successful sites was approximately 0.7 cm^2^. CS, coronary sinus; LA, left atrium; LV, left ventricle; RVa, right ventricular apex.

**FIGURE 3:**
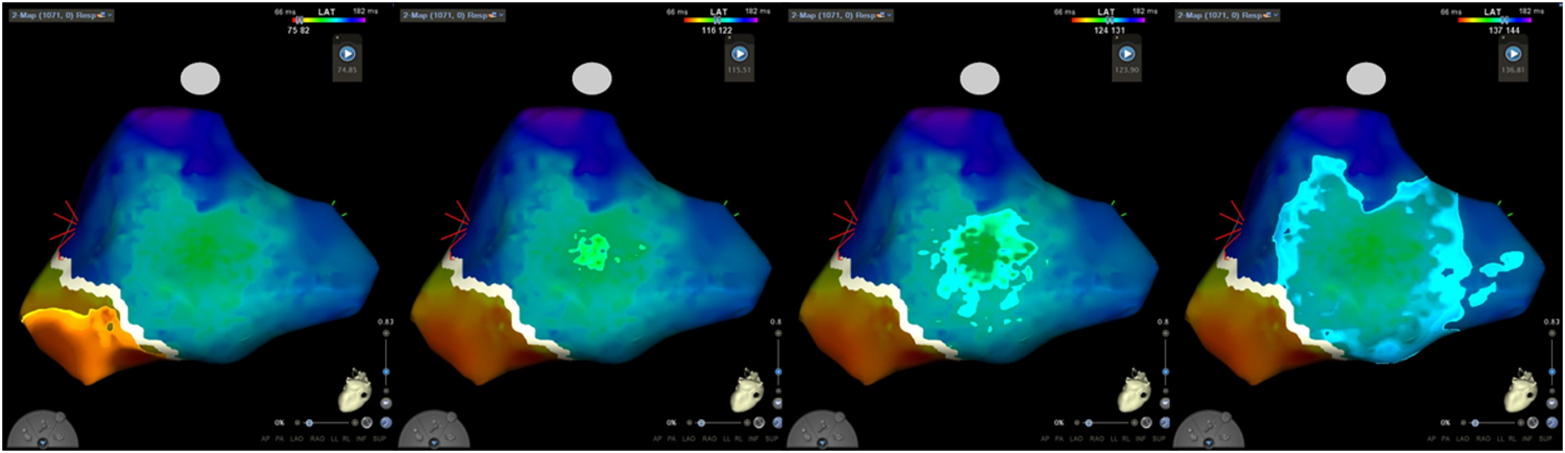
Propagation map during right ventricular apex pacing showed that after the ventricular excitation conducted to the mitral annulus (MA), the excitation temporarily disappeared and propagated as if it was springing from the left atrial posterolateral wall 2 cm above the MA.

OWM is a useful mapping technique that reduces annotation errors and facilitates AP location because the near‐field potentials are obtained regardless of their origin.^1^ Visualization of APs using OWM with EML has been reported.^2^ However, it is equally useful for the diagnosis of accessory pathways that are located away from the AV annulus as in this case.

Anatomically, APs exist within the AV groove subepicardial fat and they may course at a variable depth from subepicardial to subendocardial.^3^ In cases with atrial insertion remote from the annulus, the AP body itself crossing the annulus would also be expected to be at a distance from the annulus. It would have been difficult to eliminate AP according to the MA with conventional EPS‐based mapping. Since multiple ablations to a wide area were required, a slightly wider connection was assumed between the AP and the left atrium on the epicardial side.

There have been a few reports of left‐sided APs located away from the MA. Long DY et al. reported 5 patients who had atrial insertion of AP at the base of the left atrial appendage and 2 patients at the anterior roof of the left atrium.^4^ In all these patients, ablation to the atrial insertion site successfully abolished AP conduction. Hwang C et al. reported 4 case series of left‐free wall APs that were connected to the Marshall bundle.^5^ These were successfully eliminated by endocardial radiofrequency application toward the vein of Marshall (VOM) marked by insertion of a microcatheter or CS angiography. The success sites were reported to be the left atrial free wall 1 cm above the MA.

In the present case, AP atrial attachment was located at the left free wall 2 cm above the MA, and atrial excitation on the coronary sinus (CS) catheter was delayed, which means AP was not attached to the MA or CS musculature. Although intra‐cardiac echocardiography was not performed, it may give us more detailed information on the MA and the EAAS. In view of the successful site, the possibility of a connection between the AP and the Marshall bundle cannot be ruled out.

EML correctly drew the continuity of the white line. The advantage of using the OWM method is that the AP location can be easily visualized even in challenging cases with conventional methods, not only multiple APs, and wide AP, but also the AP located away from the AV annulus.

## FUNDING INFORMATION

This report was not supported by any grant or company.

## CONFLICT OF INTEREST STATEMENT

The authors have no conflict of interest to declare.

## PATIENT CONSENT STATEMENT

The patient has provided consent for publication.

## PERMISSION TO REPRODUCE MATERIAL FROM OTHER SOURCES

Not applicable.

## CLINICAL TRIAL REGISTRATION

None.

## Supporting information


Movie S1.

## Data Availability

Data sharing is not applicable to this article as no new data were created or analyzed in this study.
