# Supplementary figures and images for: A visualized left‐sided accessory pathway away from the mitral annulus using open window mapping with the early meets late algorithm
Source: J Arrhythm. 2023 Sep 4;39(5):822–5. doi: 10.1002/joa3.12910 (PMC10549864; doi:10.1002/joa3.12910)

## Slide 1
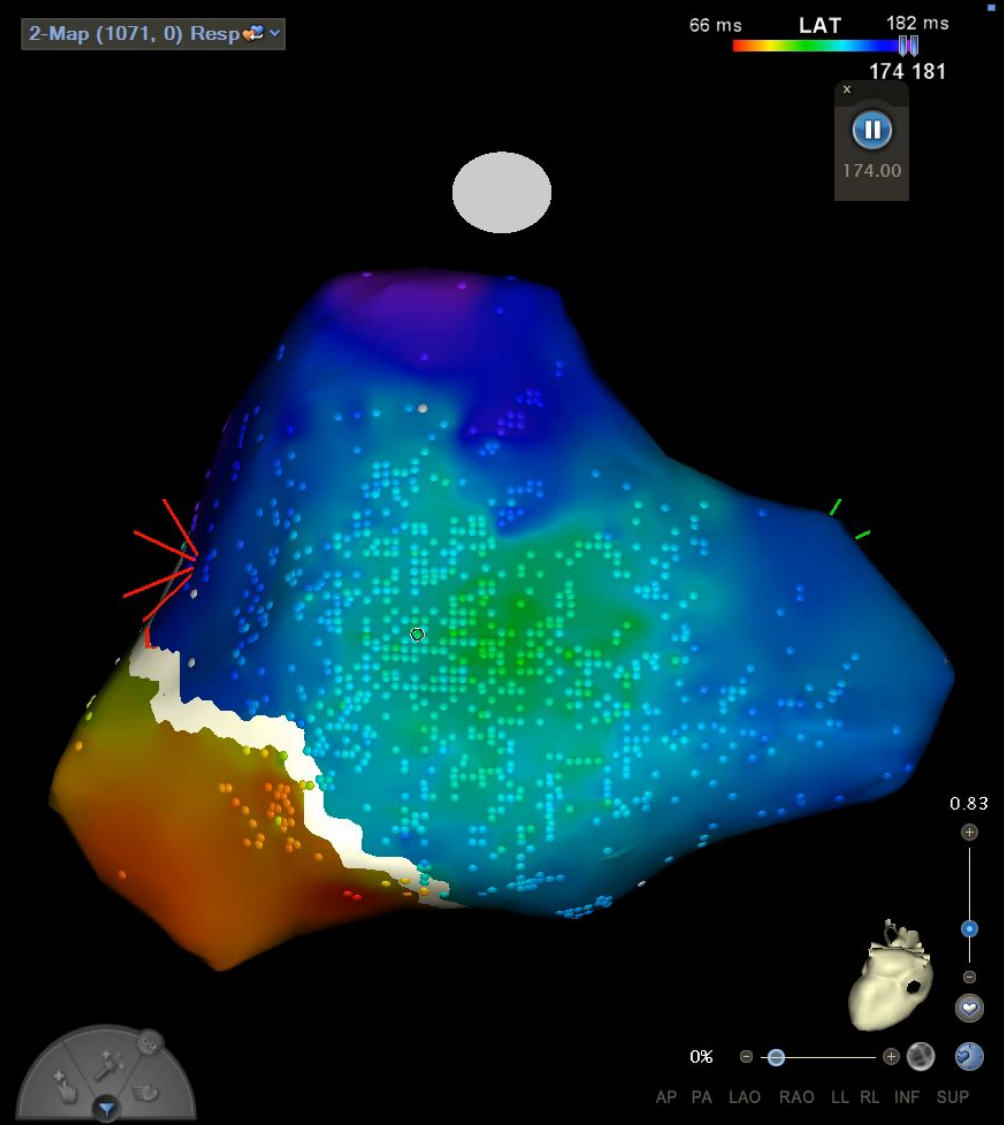

Supplement: Supplementary file 1 — Movie S1. [file JOA3-39-822-s001.pptx]
